# Machine Learning–Based Risk Factor Analysis and Prediction Model Construction for the Occurrence of Chronic Heart Failure: Health Ecologic Study

**DOI:** 10.2196/64972

**Published:** 2025-01-31

**Authors:** Qian Xu, Xue Cai, Ruicong Yu, Yueyue Zheng, Guanjie Chen, Hui Sun, Tianyun Gao, Cuirong Xu, Jing Sun

**Affiliations:** 1 School of Medicine Southeast University Nanjing China; 2 Department of Respiratory and Critical Care Zhongda Hospital Southeast University Nanjing China; 3 Department of Geriatrics Zhongda Hospital Southeast University Nanjing China; 4 Department of Intensive Care Zhongda Hospital Southeast University Nanjing China; 5 Department of Nursing Zhongda Hospital Southeast University Nanjing China; 6 Rural Health Research Institute Charles Sturt University Orange Australia

**Keywords:** machine learning, chronic heart failure, risk of occurrence, prediction model, health ecology

## Abstract

**Background:**

Chronic heart failure (CHF) is a serious threat to human health, with high morbidity and mortality rates, imposing a heavy burden on the health care system and society. With the abundance of medical data and the rapid development of machine learning (ML) technologies, new opportunities are provided for in-depth investigation of the mechanisms of CHF and the construction of predictive models. The introduction of health ecology research methodology enables a comprehensive dissection of CHF risk factors from a wider range of environmental, social, and individual factors. This not only helps to identify high-risk groups at an early stage but also provides a scientific basis for the development of precise prevention and intervention strategies.

**Objective:**

This study aims to use ML to construct a predictive model of the risk of occurrence of CHF and analyze the risk of CHF from a health ecology perspective.

**Methods:**

This study sourced data from the Jackson Heart Study database. Stringent data preprocessing procedures were implemented, which included meticulous management of missing values and the standardization of data. Principal component analysis and random forest (RF) were used as feature selection techniques. Subsequently, several ML models, namely decision tree, RF, extreme gradient boosting, adaptive boosting (AdaBoost), support vector machine, naive Bayes model, multilayer perceptron, and bootstrap forest, were constructed, and their performance was evaluated. The effectiveness of the models was validated through internal validation using a 10-fold cross-validation approach on the training and validation sets. In addition, the performance metrics of each model, including accuracy, precision, sensitivity, *F*_1_-score, and area under the curve (AUC), were compared. After selecting the best model, we used hyperparameter optimization to construct a better model.

**Results:**

RF-selected features (21 in total) had an average root mean square error of 0.30, outperforming principal component analysis. Synthetic Minority Oversampling Technique and Edited Nearest Neighbors showed better accuracy in data balancing. The AdaBoost model was most effective with an AUC of 0.86, accuracy of 75.30%, precision of 0.86, sensitivity of 0.69, and *F*_1_-score of 0.76. Validation on the training and validation sets through 10-fold cross-validation gave an AUC of 0.97, an accuracy of 91.27%, a precision of 0.94, a sensitivity of 0.92, and an *F*_1_-score of 0.94. After random search processing, the accuracy and AUC of AdaBoost improved. Its accuracy was 77.68% and its AUC was 0.86.

**Conclusions:**

This study offered insights into CHF risk prediction. Future research should focus on prospective studies, diverse data, advanced techniques, longitudinal studies, and exploring factor interactions for better CHF prevention and management.

## Introduction

Heart failure is a complex clinical syndrome where the ventricular filling or ejection capacity is compromised due to any structural or functional abnormality of the heart [1]. Chronic heart failure (CHF) is a severe manifestation or advanced stage of various cardiovascular diseases. It features high mortality and rehospitalization rates, thus constituting the ultimate battlefield for cardiovascular disease prevention and control [2]. According to the World Health Organization, the global prevalence of heart failure in adults is 1% to 2%, with 25,000-30,000 new cases expected each year [3]. The multiple physical and psychological symptoms endured by patients with CHF not only intensify the burden of the patients and caregivers but also diminish their quality of life [4,5]. Therefore, it has become an inevitable trend to identify risk factors for those who have not developed CHF and patients with CHF, to prevent the occurrence of CHF in a timely manner, and to strengthen the health management of patients with CHF.

Preventive management in current guidelines and practice is mostly based on comprehensive cardiovascular interventions [6]. However, within the framework of the goals of “health for all” and “personalized medicine,” early and precise risk stratification takes precedence in cardiovascular prevention and control [7]. Risk factors for heart failure are the primary concern in suspected diagnoses, and the control of risk factors is the top priority in the primary prevention of patients with heart failure. Diagnosis, treatment, and management are based on this. Risk assessment, as the initiating link of precise health management of CHF, particularly needs to recognize the significance of risk factor identification in the face of the disease characteristics of CHF, such as complex etiology, severe condition, rapid progression, and poor prognosis. With the in-depth research of precision medicine and the change in residents’ living behaviors, the update of important risk factors and cardiovascular markers is a crucial element in the development of risk assessment programs and the improvement of assessment tools.

The accuracy and comprehensiveness of identifying the risk factors of CHF, as well as the advancement and scientific nature of the modeling techniques, are the keys to guaranteeing the smooth implementation of risk assessment. As for the risk factors, they mainly include personal factors (such as gender, age, weight, etc) [8] and disease-related factors (such as ejection fraction, biomarkers, myocardial imaging, cardiac ultrasound parameters, etc). For instance, Salvioni et al [9] developed a method for integrating Metabolic Exercise Test data combined with Cardiac and Kidney Indexes (MECKI) scores from 2715 patients. It was demonstrated that the MECKI score is a highly efficient tool for facilitating risk stratification and therapeutic decision-making for patients with heart failure. Klimczak-Tomaniak et al [10] conducted repeated measurements of 92 biomarkers that optimally predict adverse clinical events in heart failure and can be used for dynamic risk assessment in clinical practice. However, these risk factor identifications focus only on individual and disease treatment–related factors, ignoring broader social factors that affect health. In terms of modeling techniques, traditional modeling methods such as Cox and logistic regressions cannot well deal with the complex relationships that may exist between variables, and researchers have begun to introduce digital technology into model construction to obtain a large amount of data information in the database after multidimensional interactions. Angraal et al [11] used a random forest (RF) model to predict mortality and hospitalization rates for heart failure in outpatients with preserved ejection fraction. Wang et al [12] used a machine learning model to accurately predict the risk of heart failure in older patients with prediabetes or diabetes using data from the National Health and Nutrition Examination Survey. Although risk assessment models based on big data and artificial intelligence exist, the mainstay of current risk assessment approaches remains the risk factor itself. Moreover, most of the models are predicated on the patient’s medical record information from the initial diagnosis and hospitalization, intended for medical and nursing professional evaluations, as well as checks of objective data such as those from laboratories, and a dynamic prediction model that changes with the condition has not been established.

In summary, in the face of the incomplete coverage of clinical risk factors of CHF and the low accuracy of risk prediction modeling, constructing an accurate and convenient heart failure risk prediction model is an important pathway and tool for the identification of accurate risk factors, and fully using the big data resources for the refinement of risk factors and completing the digital modeling is an important guarantee for the effectiveness of the final risk assessment.

## Methods

### Data Source

This study applied to the Jackson Heart Study (JHS) database through the National Heart Lung and Blood Institute. The database contains data on 3883 individuals. The participants were African American adults aged 35-84 years. Data were collected at baseline (V1: 2000-2004), first follow-up (V2: 2005-2008), and second follow-up (V3: 2009-2013). There were 489 patients with CHF at baseline and 3394 without CHF. The first follow-up V2 (2005-2008) lost 777 cases, leaving 3106 cases; the second follow-up V3 (2009-2013) lost 462 cases, leaving 2644 cases.

### Study Population

Patients who did not have heart failure at baseline (V1) of the JHS database were selected for this study (Textbox 1).

**Inclusion and exclusion criteria.**

**Inclusion criteria**

- Those who did not have chronic heart failure at baseline
- Those who participated in the first and second follow-up visits, or those who had electronic medical records

**Exclusion criteria**

- Patients who met the Framingham heart failure diagnostic criteria [13]

### Study Variables

The inclusion of study variables was carried out at 5 levels of health ecology. A total of 53 variables were finally included. Among them, the individual trait level, including general information, biological indicators, disease history, family history, and symptoms, had a total of 28 variables; the individual behavioral trait level, including diet, exercise, sleep, and psychology, had a total of 12 variables; the interpersonal relationship level, including family relationship, social relationship, neighborhood relationship, had a total of 4 variables; the work and life level, including working conditions, living conditions, access to health care, had a total of 8 variables; and the macropolicy level had only 1 variable, being health insurance policy. All the variables can be seen in Table S1 in Multimedia Appendix 1.

### Statistical Analyses

The study encompassed several crucial steps. In the data preprocessing stage, 2 primary operations were undertaken. First, for data proofreading, a check was made to determine if there were any missing values within the data. In the case of categorized data, values were assigned if necessary. Second, with regard to missing value processing, data with missing values exceeding 30% were directly eliminated. Concurrently, for data with missing values less than 30%, Monte Carlo multiple interpolation was used for interpolation.

Proceeding to the data analysis phase, SPSS 22.0 (IBM Corp) was used for data statistical analysis. For measurement data, if they adhered to a normal distribution, a *t* test (2-tailed) was used for comparison between groups. The *t* test (2-tailed) was a statistical test designed to determine if there was a significant difference between the means of the two groups. In the event that the measurement data did not follow a normal distribution, they were represented as median (IQR). Count data were described by frequency and percentage. Regarding the measured information in the influencing factors, if it satisfied the normal distribution, the independent samples *t* test (2-tailed) was used for testing. The independent samples *t* test (2-tailed) was a method for comparing the means of two independent groups. If the measured information in the influencing factors did not conform to the normal distribution, the Mann-Whitney *U* rank sum test was used. The Mann-Whitney *U* rank sum test was a nonparametric test used for comparing two independent groups. This study consisted entirely of count data, frequency and percentage were used for statistical description, and the chi-square test was used for statistical analysis. The chi-square test was a statistical test designed to determine if there was a significant association between two categorical variables.

Next, in the delineation of the dataset step, this study used randomized splitting to divide the dataset into a training set (70%), a test set (15%), and a validation set (15%).

In the feature selection process, before feature selection, the data were standardized. Then, after the completion of the standardization process, feature selection was carried out. Pycharm (JetBrains Corp) was used. Principal component analysis (PCA) and RF were two methods used to screen the variables, respectively. PCA was a statistical procedure that used an orthogonal transformation to convert a set of correlated variables into a set of uncorrelated variables known as principal components [14]. RF was an ensemble learning method for classification, regression, and other tasks that operated by constructing a multitude of decision trees during training and outputting the class that was the mode of the classes of the individual trees [15]. The two feature selection methods were compared, the importance of the features was ranked, and the visualization of feature importance was also performed.

In the imbalance data handling stage, this study used 5 methods for processing unbalanced datasets. These methods included oversampling, undersampling, Adaptive Synthetics Sampling, Synthetic Minority Over-sampling Technique (SMOTE), and Synthetic Minority Over-sampling Technique and Edited Nearest Neighbors (SMOTE-ENN). Models constructed using the original dataset were compared with models constructed from datasets that had been processed by these 5 methods.

Finally, in the model construction stage, 7 models were constructed in this study. They were decision tree, RF, support vector machine, extreme gradient boosting, adaptive boosting (AdaBoost), naive Bayes model, multilayer perceptron, and bootstrap forest. The decision tree was a flowchart-like structure where each internal node represented a test on an attribute, each branch represented the outcome of a test, and each leaf node represented a class or a value [16]. The RF was an ensemble learning method as previously mentioned [17]. The support vector machine was a supervised learning algorithm that analyzed data for classification and regression analysis [18]. Extreme gradient boosting was an efficient implementation of gradient boosting for large datasets [19]. AdaBoost was an iterative algorithm that combined multiple weak classifiers to form a strong classifier [20]. The naive Bayes model was a probabilistic classifier based on applying Bayes’ theorem with strong independence assumptions between the features [16]. The multilayer perceptron was a type of artificial neural network with multiple layers of neurons [21]. The bootstrap forest was an ensemble method that combined multiple bootstrap samples to build a forest of trees. The accuracy, precision, sensitivity, *F*_1_-score, and area under the curve (AUC) of each model were compared, and receiver operating characteristic (ROC) curves of each model were drawn. After selecting the optimal model, a 10-fold cross-validation was performed using the training and validation set. In order to construct a better model, we used hyperparameter optimization to find the best combination of parameters that makes the model perform best on the training set and achieve better results on the test set.

### Ethical Considerations

The study was approved by the Ethics Committee of Zhongda Hospital, Southeast University (2022ZDSYLL401-Y01).

## Results

### Patient Characteristics

**Figure 1:**
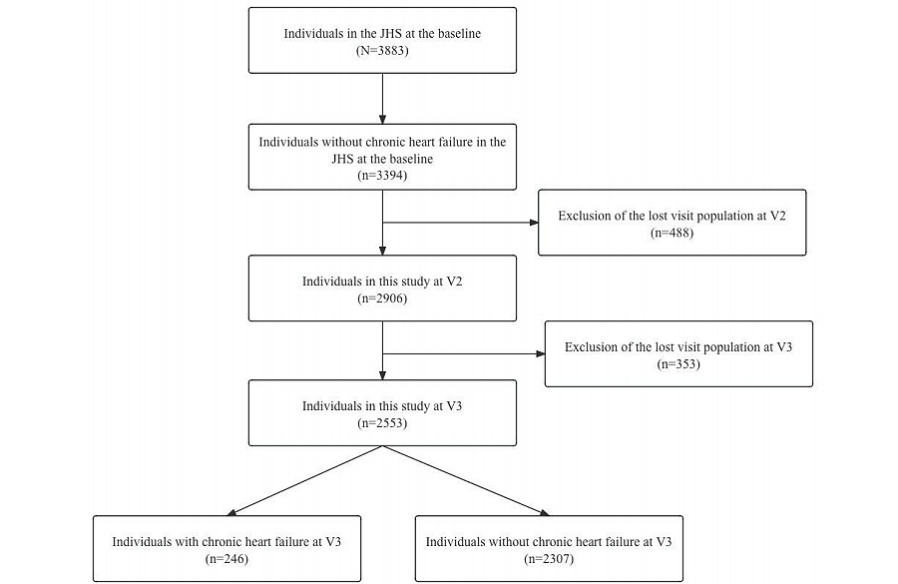
Study flowchart. CHF: chronic heart failure; JHS: Jackson Heart Study; V2: first follow-up; V3: second follow-up.

Among the 3883 individuals in the JHS, 489 had CHF at baseline and 3394 did not have CHF. Excluding patients who were lost to visit and did not have any electronic medical records, 2553 people did not have CHF at baseline. The mean age of this population was 57.84 (13.45) years, with 1590 female participants (62.2% of the population) and 963 male participants (37.7% of the population). The screening process is shown in Figure 1. The dataset (n=2553) was randomly divided into three parts: 70% (n=1787) of the data for model training, 15% (n=383) for model test and 15% (n=383) for model validation. The amount of missing data for the predictors left ventricular diastolic diameter, left ventricular systolic diameter, left ventricular mass, depression, vitamin D_2_, vitamin D_3_ derivatives, shortness of breath, walking 100 m with wheezing, and loneliness was greater than 30%, so these predictors were not included in the analysis.

### Univariate Analysis of Variables Influencing the Occurrence of CHF

Alcohol, smoking, health insurance, income, occupation, education, minutes of walking or running, nocturnal sleep dyspnea, ever had swelling of feet or ankles, chest ever sound wheezy without cold, left ventricular regional wall motion, cardiac disease, age, BMI, systolic blood pressure, glycosylated hemoglobin, triglycerides, ultrasensitive C-reactive protein, glomerular filtration rate, heart rate, stress, regional poverty population ratio, the status of favorite food stores within 3 km, status of sports facilities within 3 km, and the ratio of early maximal ventricular filling velocity to atrial maximal ventricular filling velocity were statistically significantly different in the population without heart failure (*P*<.05). A one-way analysis of the baseline characteristics of the included patients is shown in Tables 1-3.

**Table 1:** One-way analysis of the baseline characteristics (part 1).

Variables		People without heart failure (n=2307), n (%)	People with heart failure (n=264), n (%)	Chi-square (*df*)		*P*	
**Gender**					0.3 (1)		.56
	Woman	1441 (56.4)	149 (5.84)				
	Man	866 (33.92)	97 (3.8)				
**Alcohol**					26.2 (1)		<.001
	Yes	1114 (43.63)	76 (2.98)				
	No	1183 (46.34)	167 (6.54)				
**Smoking**					11.1 (1)		<.001
	Yes	680 (2.66)	98 (3.84)				
	No	1621 (63.49)	148 (5.8)				
**Health insurance**					9.5 (1)		.002
	Yes	1972 (77.24)	192 (7.52)				
	No	335 (13.12)	54 (2.12)				
**Threats or harassment**					12.1 (6)		.06
	Several times a day	15 (0.59)	3 (0.12)				
	Almost every day	15 (0.59)	3 (0.12)				
	At least once a week	45 (1.76)	3 (0.12)				
	A few times a month	42 (1.65)	4 (0.16)				
	A few times a year	102 (4)	3 (0.12)				
	Less than a few times a year	351 (13.75)	27 (1.06)				
	Never	1704 (66.75)	196 (7.68)				
**Income**					47.5 (3)		<.001
	Poor	198 (7.76)	42 (1.65)				
	Lower-middle	431 (16.88)	71 (2.78)				
	Upper-middle	632 (24.76)	56 (2.19)				
	Affluent	707 (27.69)	39 (1.53)				
**Occupation**					31.6 (10)		<.001
	Management or Professional	903 (35.37)	71 (2.78)				
	Service	515 (20.17)	81 (3.17)				
	Sales	441 (17.27)	30 (1.18)				
	Farming	2 (0.08)	0 (0)				
	Construction	119 (4.66)	19 (0.74)				
	Production	311 (12.18)	44 (1.72)				
	Military	3 (0.12)	0 (0)				
	Sick	2 (0.08)	0 (0)				
	Unemployed	2 (0.08)	1 (0.04)				
	Retired	3 (0.12)	0 (0)				
	Student	2 (0.08)	0 (0)				
**Education**					100.5 (2)		<.001
	Less than high school	300 (11.75)	91 (3.56)				
	High-school graduate or General Educational Development	459 (17.98)	45 (1.76)				
	Attended vocational school, trade school, or college	1543 (60.43)	110 (4.31)				

**Table 2:** One-way analysis of the baseline characteristics (part 2).

Variables		People without heart failure (n=2307), n (%)	People with heart failure (n=264), n (%)	Chi-square (*df*)		*P*	
**Stress living in neighborhood**					2.1 (3)		.56
	Not stressful	1708 (6.66)	188 (7.36)				
	Mildly stressful	348 (13.63)	31 (1.21)				
	Moderately stressful	148 (5.80)	12 (0.59)				
	Very stressful	91 (3.56)	11 (4.31)				
**Minutes of walking or running**					10.1 (4)		.04
	Less than 5 minutes	1069 (41.87)	133 (5.21)				
	At least 5 but less than 15 minutes	405 (15.87)	44 (1.72)				
	At least 15 but less than 30 minutes	331 (12.97)	22 (0.86)				
	At least 30 but less than 45 minutes	200 (7.83)	24 (0.94)				
	At least 45 minutes	298 (11.67)	23 (0.90)				
**Place they usually go to for health care**					11.4 (8)		.18
	Walk-in clinic	180 (7.05)	11 (0.43)				
	Health Maintenance Organization clinic	16 (0.63)	0 (0)				
	Hospital clinic	242 (9.48)	30 (1.18)				
	Neighborhood health center	115 (4.5)	18 (0.71)				
	Hospital emergency room	66 (2.59)	10 (0.39)				
	Public health department clinic	23 (0.9)	2 (0.08)				
	Company or industry clinic	63 (2.47)	4 (0.16)				
	Doctor’s office	1334 (52.25)	155 (6.07)				
	Other	12 (0.47)	0 (0)				
**Difficulty in obtaining health service**					6.2 (3)		.10
	Very hard	85 (3.33)	13 (0.51)				
	Fairly hard	114 (4.47)	20 (0.78)				
	Not too hard	395 (15.47)	42 (1.65)				
	Not hard at all	1694 (66.35)	171 (6.7)				
**Satisfied with doctor**					1.7 (4)		.79
	Very satisfied	1524 (59.69)	173 (6.78)				
	Somewhat satisfied	595 (23.31)	60 (2.35)				
	Somewhat dissatisfied	56 (2.19)	5 (0.2)				
	Very dissatisfied	21 (0.82)	2 (0.08)				
	Not sure	38 (1.49)	3 (0.12)				
**Ever awakened by trouble breathing**					102.4 (1)		<.001
	Yes	78 (3.06)	44 (1.72)				
	No	2204 (86.33)	200 (7.83)				
**Rate your sleep quality overall**					7.5 (4)		.68
	Excellent	232 (9.09)	23 (0.9)				
	Fair	514 (20.13)	52 (2.04)				
	Good	796 (31.18)	77 (3.02)				
	Poor	190 (7.44)	31 (1.21)				
	Very good	562 (22.01)	52 (2.04)				
**Ever had swelling of feet or ankles**					27.1 (1)		<.001
	Yes	956 (37.45)	144 (5.64)				
	No	1328 (52.02)	99 (3.88)				
**Chest ever sound wheezy without cold**					21.8 (1)		<.001
	Yes	156 (6.11)	37 (1.45)				
	No	2131 (83.47)	207 (8.11)				
**Marriage**					37.5 (4)		<.001
	Divorced	342 (13.4)	31 (1.21)				
	Married	1356 (53.11)	118 (4.62)				
	Unmarried	258 (10.11)	26 (1.02)				
	Separate	92 (3.6)	11 (0.43)				
	Widowed	249 (9.75)	59 (2.31)				
**LV^a^ regional wall motion**					29.7 (3)		<.001
	Abnormal	7 (2.74)	4 (0.16)				
	Border	7 (2.74)	6 (0.24)				
	Normal	2179 (85.35)	222 (8.7)				
	Can’t assess	13 (0.51)	1 (0.04)				
**Cardiac disease**					66.6 (1)		<.001
	Yes	168 (6.58)	56 (2.19)				
	No	2139 (83.78)	190 (7.44)				
**Family history of cardiovascular disease, n (%)**					0.0 (1)		.87
	Yes	748 (29.30)	81 (3.17)				
	No	1559 (61.07)	165 (6.46)				

^a^LV: left ventricle.

**Table 3:** One-way analysis of the baseline characteristics (part 3).

Variables	People without heart failure, median (IQR)	People with heart failure, median (IQR)	Mann-Whitney *U* test	*P*
Age (years)	54 (45-63)	67 (58.75-73)	–11.9	
BMI (kg/m^2^)	30.29 (26.74-35)	31.58 (28.06-42.61)	–3.59	<.001
Systolic blood pressure (mm Hg)	123.83 (114.66-134.83)	132.08 (122.91-145.38)	–7.83	<.001
Glycosylated hemoglobin (%)	5.6 (5.3-6.1)	6 (5.5-7.3)	–7.39	<.001
Low-density lipoprotein (mg/dL)	126 (102-149)	120.5 (99.75-147.25)^c^	–1.16	.25
High-density lipoprotein (mg/dL)	50 (42-60)	49 (40-58)^c^	–1.66	.10
Fasting triglycerides (mg/dL)	87 (63-124)	98 (74-141)^c^	–4.35	<.001
Fasting total cholesterol (mg/dL)	197 (173-223)	193 (170-220)^c^	–0.97	.33
Ultrasensitive C-Reactive protein (mg/dL)	0.25 (0.1-0.54)	0.31 (0.14-0.65)	–2.73	.01
Glomerular filtration rate (mL/min)	87.09 (76.97-97.78)	80.16 (64.43-91.9)	–6.65	<.001
Ejection fraction (%)	65 (55-65)	65 (55-65)	–1.18	.24
Heart rate (beats/min)	63 (56-70)	66 (57-73)	–3.37	<.001
Vitamin D_3_ (ng/mL)	12.2 (8.7-16.9)	11.7 (8.75-15.45)	–1.29	.20
Dark-colored green vegetables (ng/mL)	0.26 (0.15-0.42)	0.24 (0.15-0.39)	–1.14	.25
Stress	4 (2-8)	4 (1-7)	–2.69	.01
Egg (ng/mL)	0.32 (0.91-0.66)	0.32 (0.84-0.8)	–0.07	.94
Fish (ng/mL)	0.86 (0-0.17)	0.86 (0-0.2)	–1.53	.13
Proportion of the population living in poverty in the area (%)	0.22 (0.1-0.32)	0.31 (0.21-0.36)	–5.24	<.001
Status of favorite food stores within 3 km	0.24 (0.06-0.44)	0.34 (0.16-0.49)	–4.43	<.001
Status of sports facilities within 3 km	0.37 (0.18-0.63)	0.4 (0.23-0.71)	–2.24	.03
Peak early diastolic velocity of mitral annulus (cm/s)	0.83 (0.71-0.96)	0.8 (0.67-0.97)	–1.10	.27
Ratio of early maximal ventricular filling velocity to atrial maximal ventricular filling velocity	1.06 (0.87-1.27)	0.92 (0.76-1.35)	–6.37	<.001
Hours of actual sleep at night	6 (6-7)	6 (5-8)	–1.04	.30

### Machine Learning Analysis of the Occurrence of CHF

#### Feature Selection

This study made use of PCA and the RF method for feature selection. The model was trained using root mean square error (RMSE) as a criterion. The principle is that each step includes an additional feature with the highest variance as a basis for classification [22]. The number of selected features was used as the horizontal axis and the predicted RMSE score for each fitted model was used as the vertical axis.

**Figure 2:**
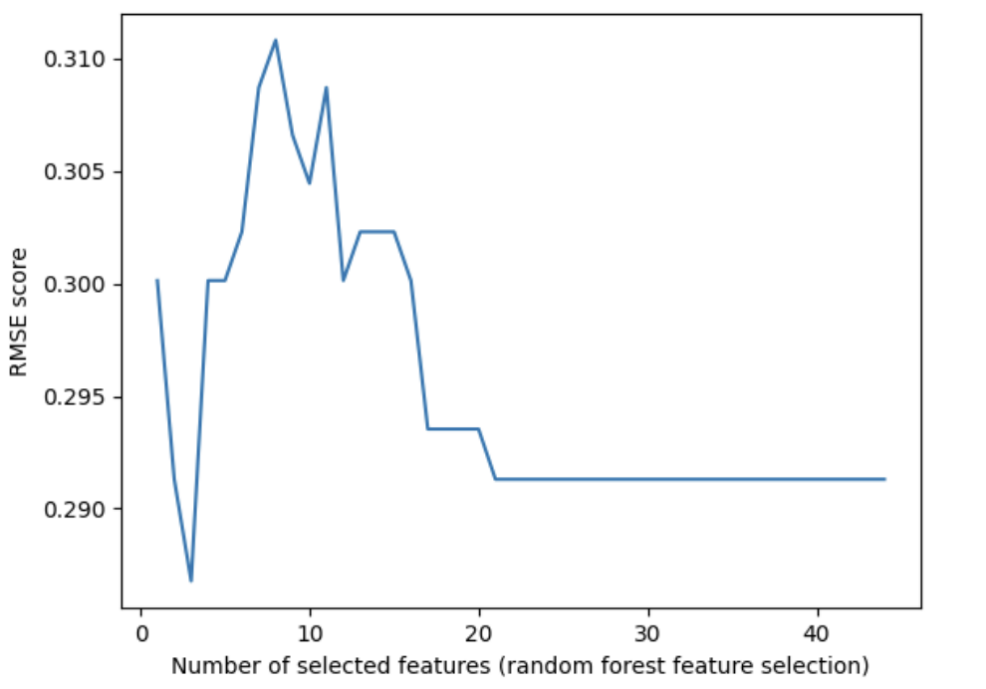
Root mean square error (RMSE) results of the principal component analysis.

PCA offers the advantage of transforming a set of correlated variables into a set of uncorrelated principal components, thereby reducing dimensionality and enhancing interpretability. After feature selection by PCA, a total of 15 features were incorporated. The initial eigenvalues, percentage of variance, and accumulation of these features are presented in Table 4. RMSE results are presented in Figure 2.

**Table 4:** The result of these features selected by principal component analysis.

Feature	Initial eigenvalue	Percentage of variance (%)	Accumulation (%)
Age (years)	3.27	7.42	7.42
Gender	2.47	5.61	13.03
Fasting total cholesterol (mg/dL)	2.21	5.02	18.04
High-density lipoprotein (mg/dL)	1.95	4.44	22.48
Status of favorite food stores within 3 km	1.78	4.03	26.52
Income	1.72	3.90	30.41
Peak early diastolic velocity of mitral annulus	1.48	3.36	33.77
LV^a^ regional wall motion	1.36	3.09	36.86
Smoke	1.26	2.85	39.71
Dark-colored green vegetables	1.22	2.78	42.49
Minutes of walking or running	1.17	2.67	45.16
Cardiac disease	1.11	2.52	47.68
Threats or harassment	1.08	2.45	50.13
Rate your sleep quality overall	1.05	2.4	52.54
Systolic blood pressure (mm Hg)	1.03	74	54.88

^a^LV: left ventricle.

**Figure 3:**
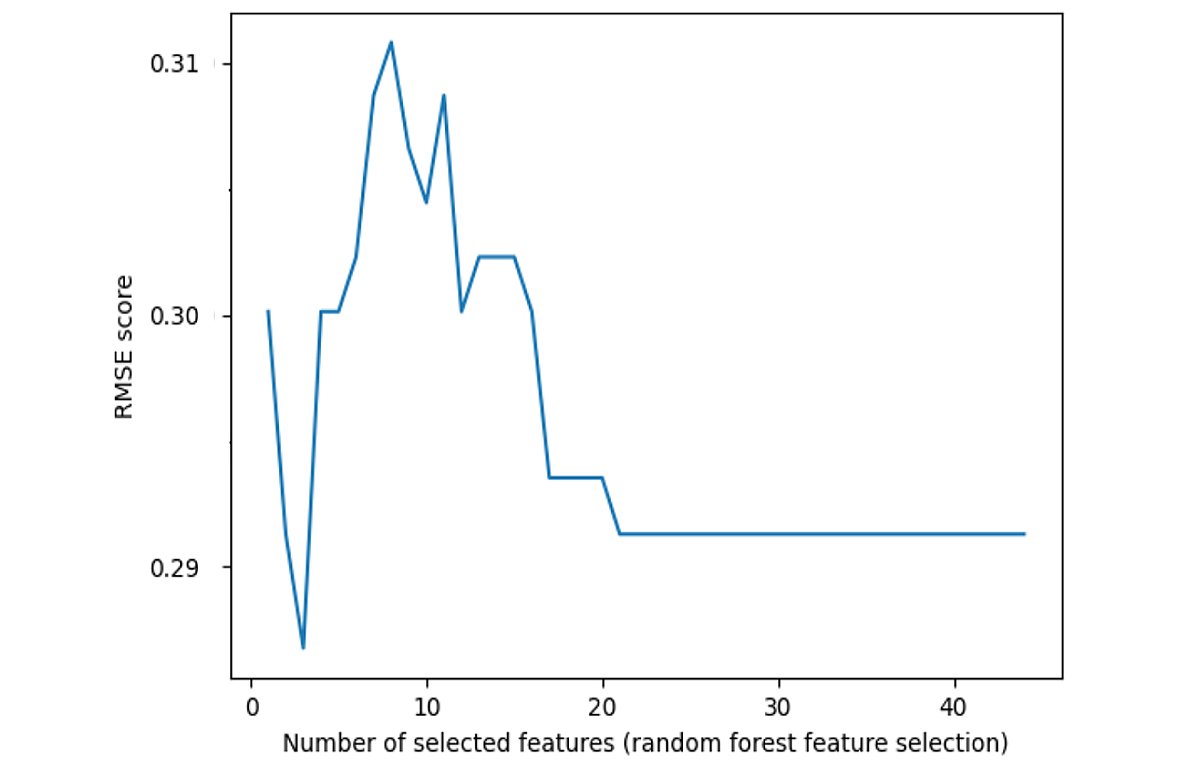
Root mean square error (RMSE) results of the random forest method.

On the other hand, the RF method is known for its robustness and ability to handle high-dimensional data. After feature selection by RF, a total of 21 features were included. The importance of these features is shown in Table 5. RMSE results are presented in Figure 3.

**Table 5:** Characteristic importance of feathers selected by random forest.

Feature	Characteristic importance
Age	0.07
Glomerular filtration rate	0.07
Glycosylated hemoglobin	0.06
Systolic blood pressure	0.05
BMI	0.04
Ratio of early maximal ventricular filling velocity to atrial maximal ventricular filling velocity	0.04
Eggs	0.04
Dark-colored green vegetables	0.04
Heart rate	0.03
Peak early diastolic velocity of mitral annulus	0.03
Fasting total cholesterol	0.03
Vitamin D_3_	0.03
Proportion of the population living in poverty in the area	0.03
Ultrasensitive C-reactive protein	0.03
Status of sports facilities within 3 km	0.03
Low-density lipoprotein	0.03
Ever awakened by trouble breathing	0.03
Status of favorite food stores within 3 km	0.03
Fasting triglycerides	0.03
High-density lipoprotein	0.03
Ejection fraction	0.03

**Figure 4:**
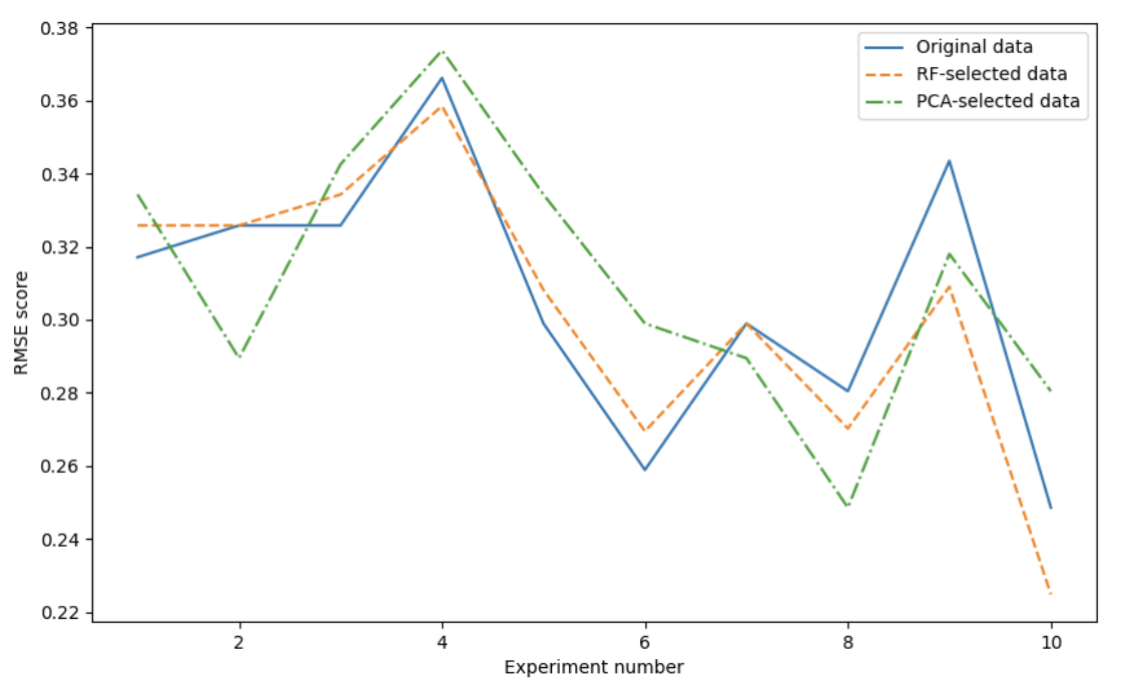
Root mean square error (RMSE) results of the original data and different feather selection. PCA: principal component analysis; RF: random forest.

To compare the feature selection methods, this study used 10-fold cross-validation. After calculating the mean RMSE and plotting the image as shown in Figure 4, the results demonstrated that the outcomes after feature selection by RF outperformed those after feature selection by PCA. The average RMSE mean of RF was 0.30. The average RMSE mean of both RF and original data was 0.31. This highlights the superiority of the RF method in terms of providing more accurate and reliable feature selection results.

#### Data Balance

This study’s analysis of machine learning algorithms using diverse data balancing techniques is presented in Table 6. The result indicated that, in contrast to other data balancing strategies used in this study, SMOTE-ENN consistently surpassed all evaluated models in terms of accuracy.

**Table 6:** Comparison of imbalanced data handling techniques across each machine learning algorithm.

Algorithms and performance metrics			Unbalanced data		Undersampling		Oversampling		ADASYN^a^		SMOTE^b^		SMOTE-ENN^c^
**Decision tree**													
	Accuracy	88.51%		56.06%		55.43%		59.30%		61.86%		68.14%	
	AUC^d^	0.66		0.56		0.55		0.60		0.62		0.71	
**Random forest**													
	Accuracy	91.38%		68.18%		54.43%		55.06%		67.86%		69.33%	
	AUC	0.81		0.83		0.77		0.80		0.80		0.85	
**XGBoost^e^**													
	Accuracy	90.86%		69.70%		59.71%		59.44%		70.57%		68.68%	
	AUC	0.80		0.76		0.77		0.81		0.79		0.81	
**AdaBoost^f^**													
	Accuracy	87.73%		71.21%		67.43%		76.76%		71.71%		75.30%	
	AUC	0.67		0.83		0.68		0.85		0.82		0.86	
**SVM^g^**													
	Accuracy	91.38%		72.73%		63.86%		63.24%		62.71%		66.78%	
	AUC	0.71		0.80		070		0.66		0.67		0.75	
**NBM^h^**													
	Accuracy	86.42%		78.79%		74.00%		72.11%		73%		77.17%	
	AUC	0.78		0.84		0.79		0.76		0.75		0.80	
**MLP^i^**													
	Accuracy	87.73%		65.16%		56%		59.15%		58.86%		62.86%	
	AUC	0.61		0.66		0.63		0.67		0.66		0.71	

^a^ADASYN: adaptive synthetics sampling.

^b^SMOTE: Synthetic Minority Oversampling Technique.

^c^SMOTE-ENN: Synthetic Minority Oversampling Technique and Edited Nearest Neighbors.

^d^AUC: area under the curve.

^e^XGBoost: extreme gradient boosting.

^f^AdaBoost: adaptive boosting.

^g^SVM: support vector machine.

^h^NBM: naïve Bayes model.

^i^MLP: multilayer perceptron.

#### Development and Performance Comparisons of Machine Learning Models

**Figure 5:**
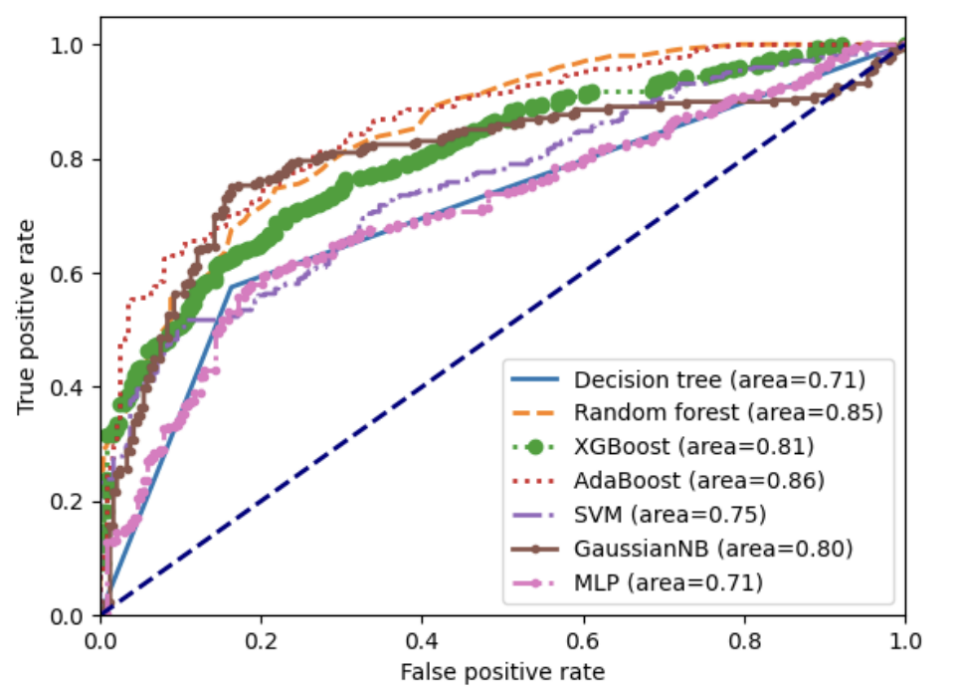
Comparison of ROC curves of different models. AdaBoost: adaptive boosting; GaussianNB: Gaussian naïve Bayes; MLP: multilayer perceptron; ROC: receiver operating characteristic; SVC: support vector classifier; XGBoost: extreme gradient boosting.

**Figure 6:**
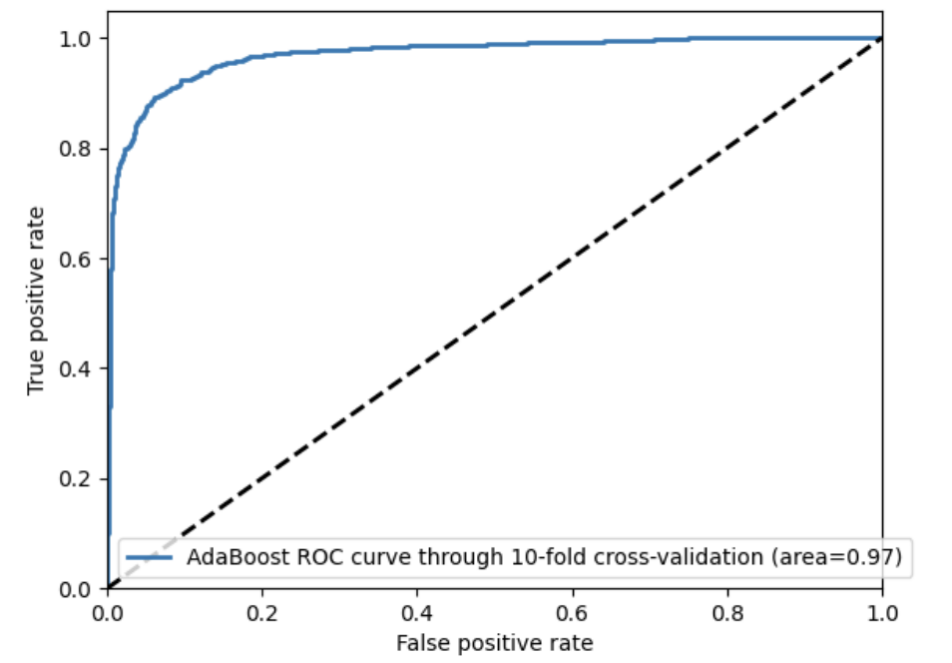
ROC curves of AdaBoost on the whole data through 10-fold cross-validation. AdaBoost: adaptive boosting; ROC: receiver operating characteristic.

This study used SMOTE-ENN for oversampling and model construction. The evaluation of each performance metric of the model is presented in Table 7, and the ROC curve of the model is shown in Figure 5. Among the models, the AdaBoost model exhibited the greatest effectiveness, boasting an AUC of 0.86, an accuracy of 75.3%, a precision of 0.86, a sensitivity of 0.69, and an *F*_1_-score of 0.76. Simultaneously, to validate the model’s efficacy, the training and validation data in this study were used to carry out validation of the model through 10-fold cross-validation. The results showed an AUC of 0.97, an accuracy of 91.27%, a precision of 0.94, a sensitivity of 0.92, an *F*_1_-score of 0.94, and the ROC curve is presented in Figure 6. We used grid search and random search on the test set to make a better model. Comparing these two methods, random search performed better on the AdaBoost model. The data processed by grid search had an accuracy of 74.79% with an AUC of 0.84. After random search processing, the accuracy and AUC of AdaBoost improved. Its accuracy was 77.68% and its AUC was 0.86.

**Table 7:** Performance comparison of different models.

Algorithms	AUC^a^ (95% CI)	Accuracy	Precision	Sensitivity	*F*_1_-score
Decision tree	0.71 (0.7-0.71)	68.14%	0.84	0.57	0.68
Random forest	0.85 (0.84-0.85)	69.33%	0.90	0.54	0.68
XGBoost^b^	0.81 (0.8-0.81)	68.68%	0.87	0.57	0.69
AdaBoost^c^	0.86 (0.85-0.86)	75.30%	0.86	0.69	0.77
SVM^d^	0.75 (0.75-0.76)	66.78%	0.87	0.52	0.65
NBM^e^	0.80 (0.79-0.8)	77.17%	0.83	0.77	0.8
MLP^f^	0.71 (0.7-0.71)	62.86%	0.83	0.47	0.6

^a^AUC: area under the curve.

^b^XGBoost: extreme gradient boosting.

^c^AdaBoost: adaptive boosting.

^d^SVM: support vector machine.

^e^NBM: naïve Bayes model.

^f^MLP: multilayer perceptron.

## Discussion

### Analysis of Risk Factor Analysis for the Occurrence of CHF Based on the Health Ecology

This study found that 22 risk factors predicting the occurrence of CHF were identified through a machine learning algorithm and hierarchically classified in accordance with the health ecology theory, with the objective of offering a comprehensive perspective for the prevention and intervention of CHF in the future. Health ecology not only takes into account the influence of individual disease factors on health but also conducts an all-round and holistic management of health from aspects such as individual traits, behavior and lifestyle, family and social interpersonal networks, and macropolicies [23].

At the individual trait level, this study identified age, glomerular filtration rate, glycosylated hemoglobin, systolic blood pressure, BMI, the ratio of early maximal ventricular filling velocity to atrial maximal ventricular filling velocity, ever awakened by trouble breathing, fasting triglycerides, heart rate, high-density lipoprotein, peak early diastolic velocity of mitral annulus, ejection fraction, fasting total cholesterol, ultrasensitive C-reactive protein, and low-density lipoprotein, for a total of 14 risk factors. Results from existing population-based cohort studies [24] suggest that despite the lower incidence and absolute risk of heart failure in younger people compared with older people, the associations of modifiable risk factors were stronger, and attributable risks were greater in younger participants, consistent with age factors. Systolic blood pressure, glycated hemoglobin, heart rate, high-density lipoprotein, low-density lipoprotein, ejection fraction, fasting total cholesterol, and ratio of early maximal ventricular filling velocity to atrial maximal ventricular filling velocity are important indicators for evaluating cardiac systolic-diastolic function, blood glucose levels, and glomerular filtration function, along with hypertension, diabetes mellitus, and comorbidities as the traditional independent risk factors for CHF [25-27]. Ever awakened by trouble breathing is used as a typical symptom of nocturnal paroxysmal dyspnea and edema in heart failure [28,29].

At the behavioral and lifestyle level, this study identified vitamin D_3_, eggs, and dark-colored green vegetables, a total of 3 risk factors. Diet has a great influence on the development of CHF and is one of the important risk factors for the development of CHF [30].

In the living and working conditions stratum, this study identified the proportion of the population living in poverty in the area, the status of sports facilities within 3 km, and the status of favorite food stores within 3 km, for a total of 3 risk factors. The status of favorite food stores within 3 km reflects the impact of dietary nutrition in at-risk populations; nutrition has been shown to be an important factor in the prevention of heart failure [31]. The status of sports facilities within 3 km could reflect the exercise situation. Studies have shown a strong association between physical inactivity, low fitness, and heart failure and have emphasized the importance of regular physical activity in the prevention and treatment of heart failure [32], which is consistent with the exercise and activity index factors identified in this study. The proportion of the population living in poverty in the area reflects income and wealth. Yusuf et al [33] conducted a study to explore the risk factors for cardiovascular events and showed that each risk factor varied according to the economic level of the country.

The above risk factor analyses based on the health ecology perspective not only make up for the limitations of the individual factor level of CHF but also provide a researchable direction for the future health management of heart failure.

### Analysis of Prediction Model Construction for the Occurrence of CHF Based on Machine Learning

At present, traditional regression methods are mainly used in clinical practice to screen for high-risk factors of CHF, and no conventional prediction methods have been found for the occurrence of CHF. Although traditional regression methods can provide effective prediction results, they still cannot effectively solve the problem of deviation between predicted and actual values, and more advanced technologies are needed to solve it. With the advent of the big data era, the application of machine learning in the medical and health field is becoming increasingly widespread, especially in disease prediction and prognosis evaluation [34]. This study compares different methods for processing unbalanced datasets. SMOTE-ENN is the optimal processing method. SMOTE-ENN combines the SMOTE and ENN methods. SMOTE is first used to generate new synthetic samples, and then the ENN method is used to clean the synthetic dataset to remove the noisy samples and outliers to improve the quality of the dataset and the classification performance of the model [35].

This study found that AdaBoost has a better ability to handle the problem of CHF occurrence, with better performance. Many studies have shown that AdaBoost is able to achieve high accuracy in solving various classification problems [36,37]. AdaBoost focuses on hard-to-classify samples by constantly adjusting the sample weights, allowing the classifier to better learn the features of these samples, thus improving overall accuracy. Currently, AdaBoost has been used in the area of heart disease. Rath et al [38] conducted a study demonstrating that the proposed AdaBoost algorithm has been shown to have an advantage over other algorithms in terms of accuracy when solving the electrocardiogram quality assessment problem. Therefore, AdaBoost is an efficient machine learning algorithm that is particularly suitable for processing large-scale datasets. Its efficient computational performance, excellent prediction accuracy, and flexible model configuration give it natural advantages in the construction of cross-domain models in medical engineering.

### Limitations

This study has certain limitations. First, due to the limitations of retrospective research attributes, there are inevitably issues such as insufficient variable collection, missing data, and anomalies. Second, there is a lack of external datasets to further test the generalization ability of the model. Finally, due to some missing data obtained from the National Heart, Lung and Blood Institute in this research database, there is a lack of biological indicators that are highly correlated with CHF, such as N-terminal pro-B-type natriuretic peptide.

### Conclusion

In this study, we endeavored to construct a predictive model for CHF occurrence using machine learning and analyze CHF risk from a health ecology perspective. Through a series of comprehensive procedures, including data preprocessing to handle missing values and standardize data, and applying feature selection methods like PCA and RF, we identified relevant factors associated with CHF. The analysis of risk factors based on health ecology provided a holistic understanding. At the individual trait level, multiple factors were recognized, along with several at the behavioral and lifestyle level and the living and working conditions level. Among the machine learning models evaluated, the AdaBoost model showed relatively high effectiveness. However, the study had limitations. The retrospective design led to issues such as incomplete variable collection and missing data, and the lack of external datasets affected the assessment of model generalization.

For future research, prospective studies should be considered to improve data quality and collection. Incorporating more diverse data sources and advanced machine learning techniques could enhance the model’s performance and generalizability. Longitudinal studies could track the changes in risk factors over time to better understand CHF development. In addition, further exploration of the interactions between different levels of health ecology factors could provide deeper insights into CHF prevention and management strategies. By addressing these areas, future research can build on the current work and contribute to more effective approaches to dealing with CHF.

## Appendix

Multimedia Appendix 1 Predictor variables.

## Abbreviations

AdaBoost: adaptive boosting
AUC: area under the curve
CHF: chronic heart failure
JHS: Jackson Heart Study
MECKI: Metabolic Exercise Test data combined with Cardiac and Kidney Indexes
ML: machine learning
PCA: principal component analysis
RF: random forest
RMSE: root mean square error
SMOTE-ENN: Synthetic Minority Oversampling Technique and Edited Nearest Neighbors
