# Supplementary material for: Machine Learning–Based Risk Factor Analysis and Prediction Model Construction for the Occurrence of Chronic Heart Failure: Health Ecologic Study
Source: JMIR Med Inform. 2025 Jan 31;13:e64972. doi: 10.2196/64972 (PMC11829185; doi:10.2196/64972)
Supplement: Multimedia Appendix 1 [file medinform_v13i1e64972_app1.docx]

**Table S1.** Predictor variables.

| Level of health ecology and dimension | | Variables |
| --- | --- | --- |
| **Individual trait level** | | |
|  | General information | Age, gender, BMI, systolic pressure |
|  | Symptom | Nocturnal dyspnea, ever had swelling of feet or ankles, chest ever sound wheezy without cold, wheezing after walking 100 km |
|  | Disease history | History of coronary artery disease, history of cardiac surgery, history of dialysis |
|  | Family history | Family history of cardiovascular disease |
|  | Biological indicators | Glycosylated hemoglobin , low-density lipoprotein , high-density lipoprotein , fasting triglycerides, fasting total cholesterol, ultrasensitive C-reactive protein, glomerular filtration rate, left ventricular mass, ejection fraction, left ventricular diastolic diameter, left ventricular systolic diameter, left ventricular wall anomalous motion, Peak early diastolic velocity of mitral annulus, the ratio of early maximal ventricular filling velocity to atrial maximal ventricular filling velocity, heart rate |
| **Individual behavioral** **trait level** | | |
|  | Diet | Vitamin D2, Vitamin D3, Vitamin D3 derivatives, dark-colored green vegetables, eggs, fish |
|  | Exercise | Minutes of walking or running |
|  | Sleep | Hours of sleep at night, quality of sleep |
|  | Psychology | Depression, stress, loneliness |
| **Interpersonal relationship level** | | |
|  | Family relationship | Marriage |
|  | Social relationship | Education, Threats or harassment |
|  | Neighborhood relationship | Neighborhood stress |
| **Work and life level** | | |
|  | Work conditions | Occupation, income |
|  | Living conditions | Proportion of the population living in poverty in the area, status of favorite food stores within 3 km, status of sports facilities within 3 km |
|  | Access to health care | Level of hospital frequented, difficulties in accessing care, satisfaction with doctors |
| **Macropolicy level** | | |
|  | Health insurance policy | Availability of medical insurance |
